# Supplementary material for: The Spin-Phonon Relaxation Mechanism of Single-Molecule Magnets in the Presence of Strong Exchange Coupling
Source: ACS Cent Sci. 2025 Mar 13;11(4):550–9. doi: 10.1021/acscentsci.4c02139 (PMC12022908; doi:10.1021/acscentsci.4c02139)
Supplement: Supplementary file 1 — oc4c02139_si_001.pdf [file oc4c02139_si_001.pdf]

The spin-phonon relaxation mechanism of single-molecule magnets in the presence of strong exchange coupling

Supplementary Information

Sourav Mondal[1], Julia Netz[2], David Hunger[3], Simon Suhr[4], Biprajit Sarkar[4], Joris van Slageren[3], Andreas Köhn[2], and Alessandro Lunghi[1*]

[1] *School of Physics, AMBER and CRANN Institute, Trinity College, Dublin 2, Ireland*
[2] *Institute for Theoretical Chemistry, University of Stuttgart, Pfaffenwaldring 55, D-70569 Stuttgart, Germany*
[3] *Institute of Physical Chemistry, University of Stuttgart, Pfaffenwaldring 55, D-70569 Stuttgart, Germany and*
[4] *Institute for Chemistry and Biochemistry, Freie Universität Berlin, Fabeckstraße 34-36, 14195 Berlin, Germany*

* lunghia@tcd.ie

AB INITIO CALCULATIONS

Input for a MS-CASPT2 computation using the Molpro program package (with additional set-up of a spin-orbit CI matrix). The corresponding geometry inputs are supplied separately:

```
memory,3800,m
gthresh,energy=1e-7

gprint,orbital,civector
gdirect

ang
nosym
geometry=co2rad_cp2k.xyz
basis={
default=def2-SVP
co=def2-TZVPP
n=def2-TZVPP
}

! initial computation: CAHF for non-oxidized bridge
charge=-4

{avas,thr=0.5,nela=14,locorb=1;
wf,,1,6
center,1,3d
center,50,3d}

{df-multi,cahf
start,2110.2,set=2
cahf,7,274.1,-278.1
cahf,7,279.1,-283.1}

! actual CASSCF computation with radical bridge
charge=-3

{df-multi
occ,284;closed,270
rotate,269.1,271.1
rotate,284.1,294.1
wf,,1,7;state,4
wf,,1,5;state,8
wf,,1,3;state,8
wf,,1,1;state,8
save,cirec=5100.2} ! the four sets will be on 5100.2 5200.2 5300.2 5400.2

! PNO-CASPT2 computations
shfpt2=0.45
local,thrpno_occ=1.d-8,thrdist=1.d-6,iext=2
{pno-caspt2,h0=2,coupcor=2,cirec=5100.2,shift=shfpt2,saveheff=5417.2,maxit=100;
wf,,1,7;state,4;thresh,thrdls=1.d-8}

{pno-caspt2,h0=2,coupcor=2,cirec=5200.2,shift=shfpt2,saveheff=5415.2,maxit=100;
wf,,1,5;state,8;thresh,thrdls=1.d-8}

{pno-caspt2,h0=2,coupcor=2,cirec=5300.2,shift=shfpt2,saveheff=5413.2,maxit=100;
wf,,1,3;state,8;thresh,thrdls=1.d-8}
```

```
{pno-caspt2,h0=2,coupcor=2,cirec=5400.2,shift=shfpt2,saveheff=5411.2,maxit=100;
wf,,1,1;state,8;thresh,thrdls=1.d-8}

! compute LOP with respect to inversion center
! use HLS=1 to get output for matrix elements (for postprocessing)
{ci;core,270;hlsmat,als,5100.2,5200.2,5300.2,5400.2,heff,5417.2,5415.2,5413.2,5411.2;
expec,lop,,9.1815515,4.8918555,8.2318685;print,HLS=1,VLS=0}
```

Input for a MS-CASPT2 computation for only one Co center (for extracting g and D tensor):

```
memory,1000,m
gthresh,energy=1e-6

gprint,orbital,civector
gdirect

ang
nosym
! one co replaced by zn in this structure:
geometry=co2rad_cp2k_zn.xyz

basis={
default=def2-SVP
co=def2-TZVPP
zn=def2-TZVPP
n=def2-TZVPP
}

! reduced state (no radical bridge)
charge=-4

{avas,thr=0.5,nela=7;
wf,,1,3
center,1,3d}

{df-multi,cahf
cahf,7,279.1,-283.1}

{df-multi
occ,283;closed,278
wf,,1,1;state,40
wf,,1,3;state,10
save,cirec=5100.2} ! the two sets will be on 5100.2 and 5200.2

shfpt2=0.4
local,thrpno_occ=1.d-8,thrdist=1.d-6,iext=2
{pno-caspt2,h0=2,coupcor=2,cirec=5100.2,shift=shfpt2,saveheff=5414.2,maxit=300;
wf,,1,1;state,40;thresh,thrdls=1.d-8}

{pno-caspt2,h0=2,coupcor=2,cirec=5200.2,shift=shfpt2,saveheff=5412.2,maxit=300;
wf,,1,3;state,10;thresh,thrdls=1.d-8}

{ci;hlsmat,als,5100.2,5200.2,heff,5414.2,5412.2,aniso;
expec,lop,co;print,HLS=1,VLS=0}

{single_aniso,mltp=[4,4]}
```

TABLE S1. CASSCF energy levels (wavenumbers in cm^{-1}, relative to the lowest sextet state) from a computation using state-averaged orbitals with 20 octet, 40 sextet, 40 quartet, and 40 doublet states. Otherwise the same settings as in the inputs shown above are used. The low-energy states considered in this study are highlighted.

$M_S = 7/2$	$M_S = 5/2$	$M_S = 3/2$	$M_S = 1/2$
574.0	0.0	80.8	161.9
1283.5	490.7	407.9	325.5
1288.0	727.0	805.2	883.5
1997.9	730.6	809.0	887.5
7052.4	1203.0	1122.9	1043.4
7057.7	1207.3	1127.1	1047.3
7766.7	1458.2	1534.0	1610.2
7767.4	1919.5	1841.6	1764.1
8065.8	6637.5	6679.6	6712.0
8070.4	6644.9	6686.6	6718.5
8776.0	7003.8	6961.2	6928.7
8784.7	7009.1	6967.0	6935.1
9027.8	7370.0	7411.8	7444.7
9029.6	7373.9	7415.3	7447.8
9129.9	7689.7	7676.4	7643.5
9141.1	7691.6	7677.8	7645.3
9739.1	7718.9	7718.4	7734.9
9742.1	7719.8	7718.9	7737.2
9839.7	8026.4	7999.4	7982.3
9855.5	8028.5	8000.2	7984.6
	8421.0	8447.7	8464.5
	8422.6	8451.5	8470.1
	8678.4	8699.4	8694.5
	8679.6	8703.3	8696.7
	8721.1	8710.6	8702.0
	8737.3	8714.6	8704.7
	8741.8	8745.2	8783.3
	8744.6	8765.3	8796.6
	9027.8	9009.9	8968.9
	9028.8	9010.4	8977.5
	9072.8	9040.5	9039.7
	9090.7	9063.7	9060.4
	9407.1	9432.2	9434.4
	9412.0	9432.8	9435.1
	9450.3	9473.2	9511.9
	9475.2	9496.9	9528.8
	9738.6	9721.1	9679.7
	9742.4	9725.7	9692.6
	9783.7	9750.6	9749.5
	9805.8	9778.5	9774.7

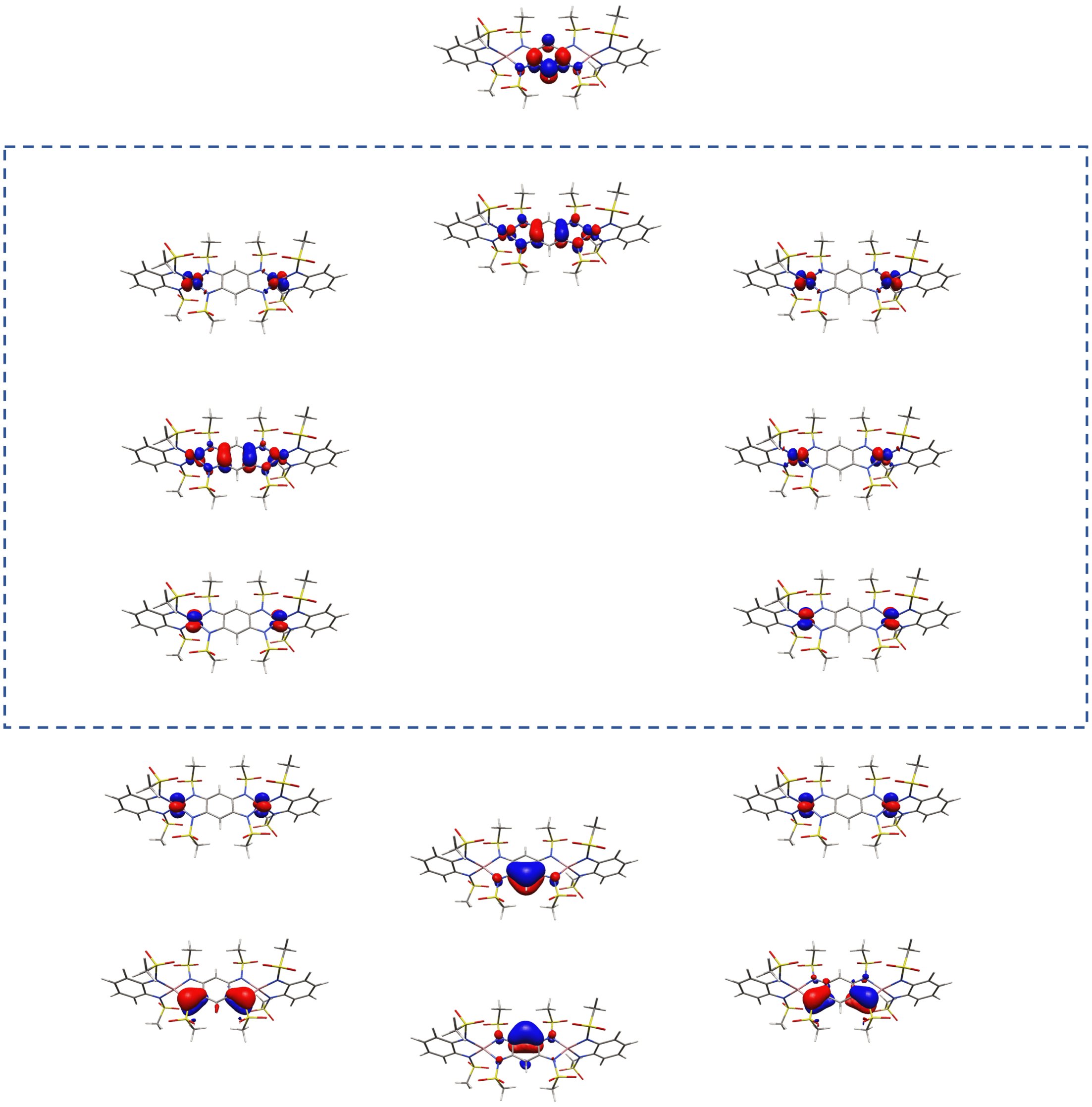

FIG. S1. **Active orbitals.** The optimized active orbitals obtained with state-averaging over 4 octet, 8 sextet, 8 quartet, and 8 doublet states. The orbitals that predominantly carry single electrons in the relevant configurations are highlighted by the dashed box.

SPIN HAMILTONIAN VALUES

The **g** tensor and the zero-field splitting (**D**) tensor are extracted from an MS-CASPT2 computation (with spin-orbit coupling added) as outlined in the previous sections. The tensors are given below, Tables S2 and S3

TABLE S2. **g** tensor for **Co₂Rad** as extracted from MS-CASPT2 calculations. The tensor is expressed in the Cartesian frame of the optimized molecular crystal cell. Eigenvalues are $g_x = 1.96$, $g_y = 2.00$, $g_z = 3.21$.

2.05845282	0.09715036	0.24583702
0.09715036	2.09994669	0.37409459
0.24583702	0.37409459	3.01234383

TABLE S3. **D** tensor (in cm^{-1}) for **Co₂Rad** as extracted from MS-CASPT2 calculations. The tensor is expressed in the Cartesian frame of the optimized molecular crystal cell. The resulting eigenvalues correspond to $D = -114.14$ cm^{-1} and $E = 0.87$ cm^{-1} and the anisotropy axes align nearly completely with the magnetic axes.

31.64412078	-8.94059442	-23.20183095
-8.94059442	26.26961239	-34.67330492
-23.20183095	-34.67330492	-57.91373317

The exchange coupling between the Co centres and the radical is extracted from the splitting between the lowest quartet and double states in the MS-CASPT2 computations, see Table S4. This choice thereby focuses on the lower part of the spin ladder, the predicted energy levels interestingly also fit well for the highest-lying octet state. The discrepancy for the three middle states cannot be resolved by a fit to the entire spectrum, adding a an exchange coupling interaction between the two Co centres does not lead to a satisfactory fit, either. While the overall RMS can be reduced, the errors for particularly the lowest states become large. The resulting value for the direct exchange coupling (ferromagnetic, > 10 cm^{-1}) appears inconsistent with the rather large distance between the nuclei. Much smaller values have been reported for dinuclear Co complexes with much short internuclear distances [1].

Anisotropic exchange contributions do only occur upon inclusion of spin-orbit or spin-spin interactions. These interaction, however, decay rapidly with the distance between the Co centres ($\propto r^{-3}$) and are not expected to exceed the 1 cm^{-1} range. The coupling between the Co centre and the radical is also not strongly altered by spin-orbit coupling effects, as our analysis of the spin-orbit coupled states does not indicate significant mixing of states with different multiplicity. Spin-spin interactions were not explicitly considered, but are known to be small even for electrons at a single centre [2]. These findings are also in line with our analysis of the analogous dinuclear Ni complex [3].

TABLE S4. **Extracting J from the computations.** All values are wavenumbers (in cm^{-1}).

	Computed	From Δ_{QD} $J = 394.48$ cm^{-1} J(Co-Co)= 0 cm^{-1}		Fit to all $J = 417.8$ cm^{-1} J(Co-Co)= 0 cm^{-1}		Fit to all $J = 405.2$ cm^{-1} J(Co-Co)= -16.5 cm^{-1}	
$2S+1$	ΔE	ΔE	δ	ΔE	δ	ΔE	δ
6	0.0	0.0		0.0		0.0	
4	198.1	197.2	-0.9	208.9	10.8	252	53.9
2	394.5	394.5	0.0	417.8	23.3	487.7	93.2
2	919.0	789.0	-130.0	835.6	-83.4	909.4	-9.6
4	1119.3	986.2	-133.1	1044.6	-74.7	1095.5	-23.8
6	1320.0	1183.4	-136.5	1253.4	-66.6	1265.1	-54.9
8	1383.5	1380.7	-2.8	1462.4	78.9	1418.17	34.6
		RMS	93.3	RMS	63.1	RMS	52.4
		RMS (lowest)	0.6	RMS (lowest)	18.2	RMS (lowest)	76.1

The full spectrum obtained by diagonalizing the spin Hamiltonian using the extracted zero-field splitting and exchange coupling parameters is represented in Fig. S2.

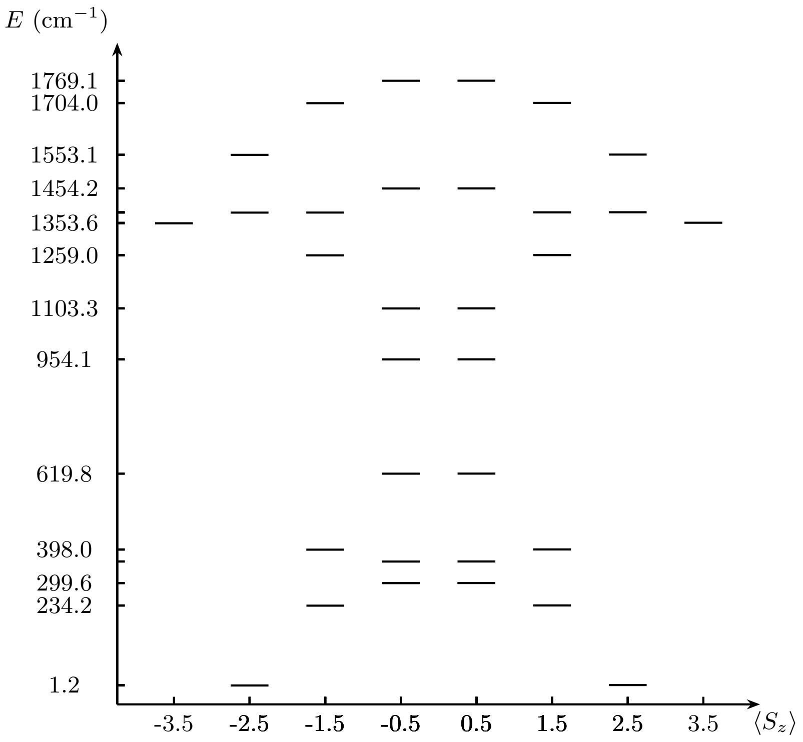

FIG. S2. **Spin energy levels of Co_2Rad.** The eigenvalues of the total spin Hamiltonian are reported as a function of the corresponding expectation values of the z-component of the total spin operator.

SUPPLEMENTARY SIMULATIONS

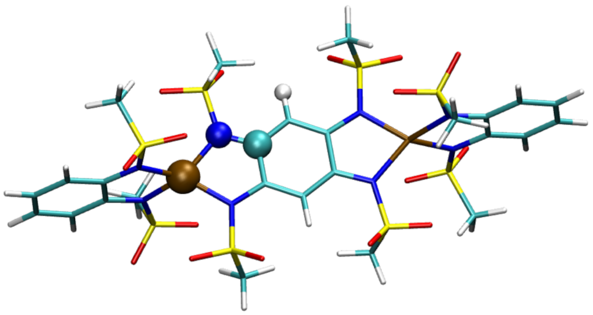

FIG. S3. **Selected atoms for the visualization of spin Hamiltonian profile.**

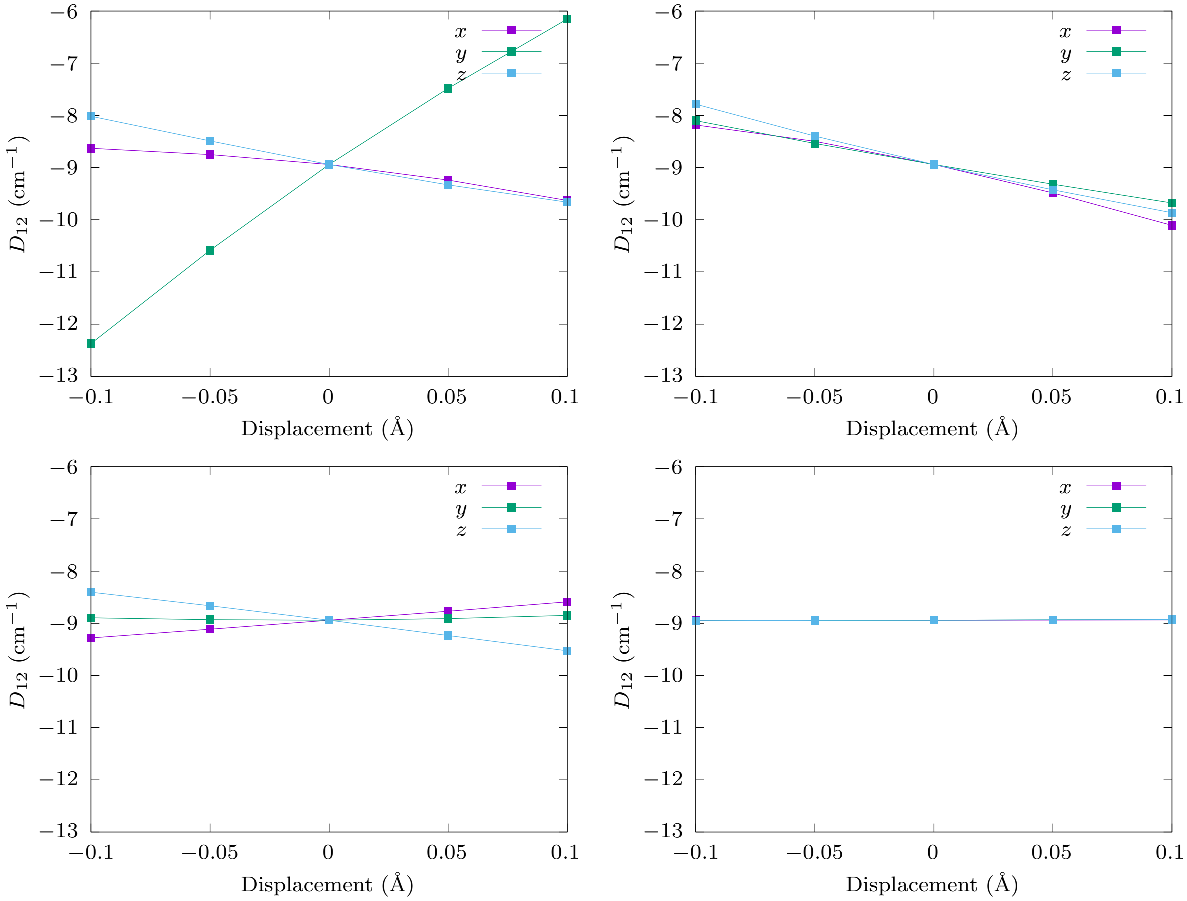

FIG. S4. **Zero-field splitting as a function of molecular distortions.** The computed value of D_{12} is reported as a function of atomic displacements for the one Co ions (top left panel), N of the bridging ligand (top right panel), C atom of the bridging ligand (bottom left panel) and H of the bridging ligand (bottom right panel). The actual position of the atoms inside the molecular structure is represented in Fig. S3.

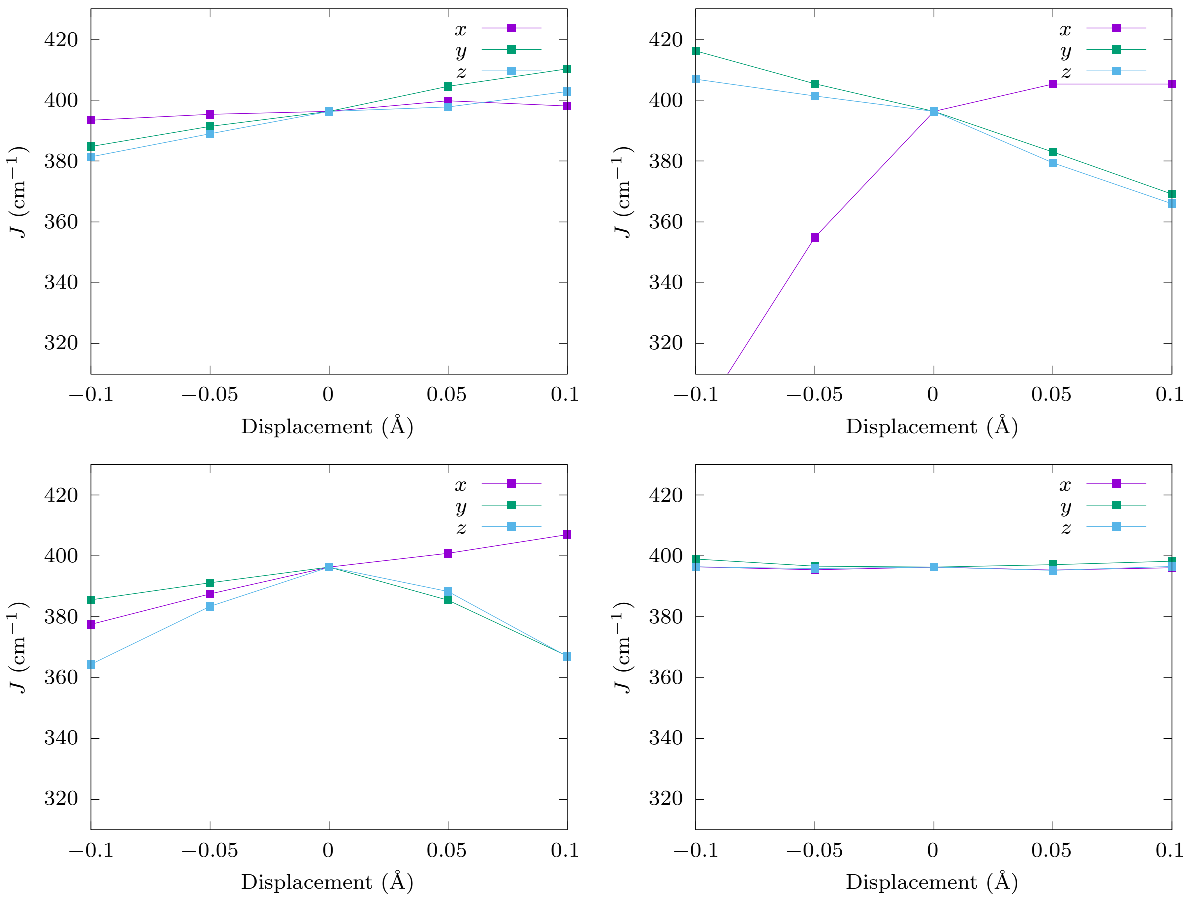

FIG. S5. **Exchange coupling as a function of molecular distortions.** The computed value of J is reported as a function of atomic displacements for the one Co ions (top left panel), N of the bridging ligand (top right panel), C atom of the bridging ligand (bottom left panel) and H of the bridging ligand (bottom right panel). The actual position of the atoms inside the molecular structure is represented in Fig. S3.

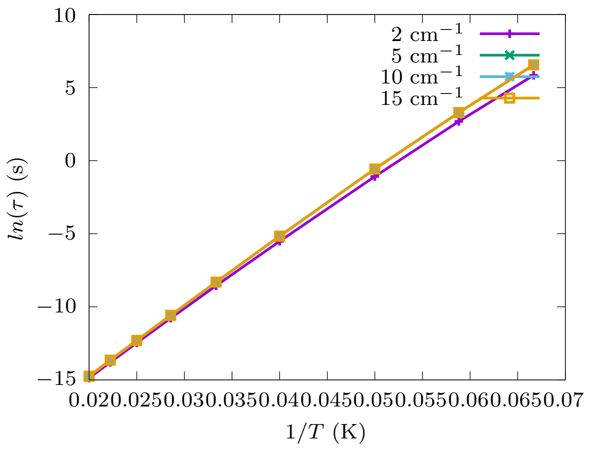

FIG. S6. **Spin relaxation time as a function of smearing.** Computed Orbach relaxation rate as a function of temperature and the smearing of the Gaussian used to represent the Dirac deltas entering the expression of W^{1-ph}.

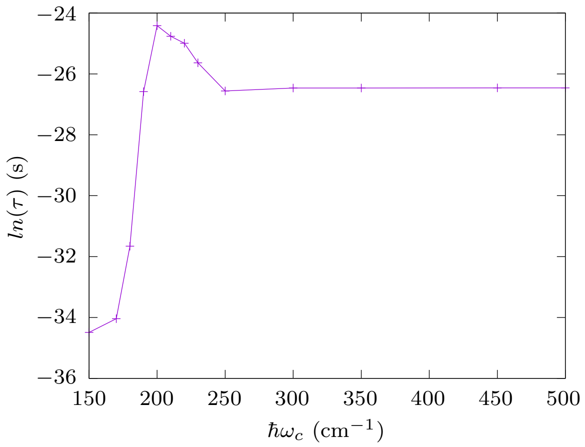

FIG. S7. **Orbach rates convergence.** Computed Orbach relaxation rate at 20 K as a function of phonons' high-energy cut-off $\hbar\omega_c$, i.e. phonons with energy higher than the cut-off are excluded from simulations.

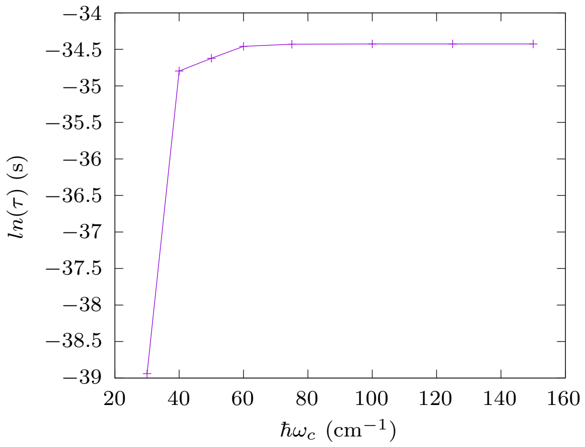

FIG. S8. **Raman rates convergence.** Computed Raman relaxation rate at 7 K as a function of phonons' high-energy cut-off $\hbar\omega_c$, i.e. phonons with energy higher than the cut-off are excluded from simulations.

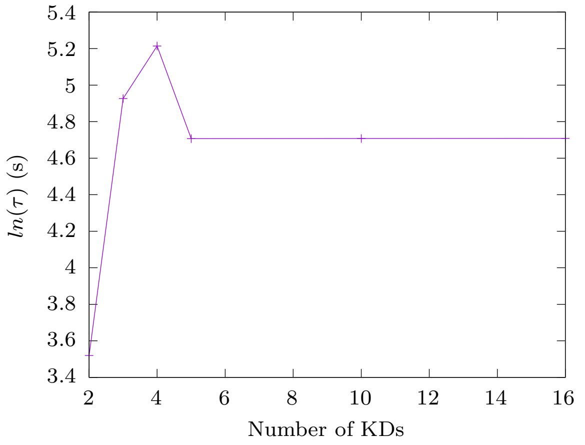

FIG. S9. **Virtual states.** Computed Raman relaxation rate at 10 K as a function of the number of KDs (including the ground-state one) included in the calculation W^{2-ph}.

COBALT CHAINS

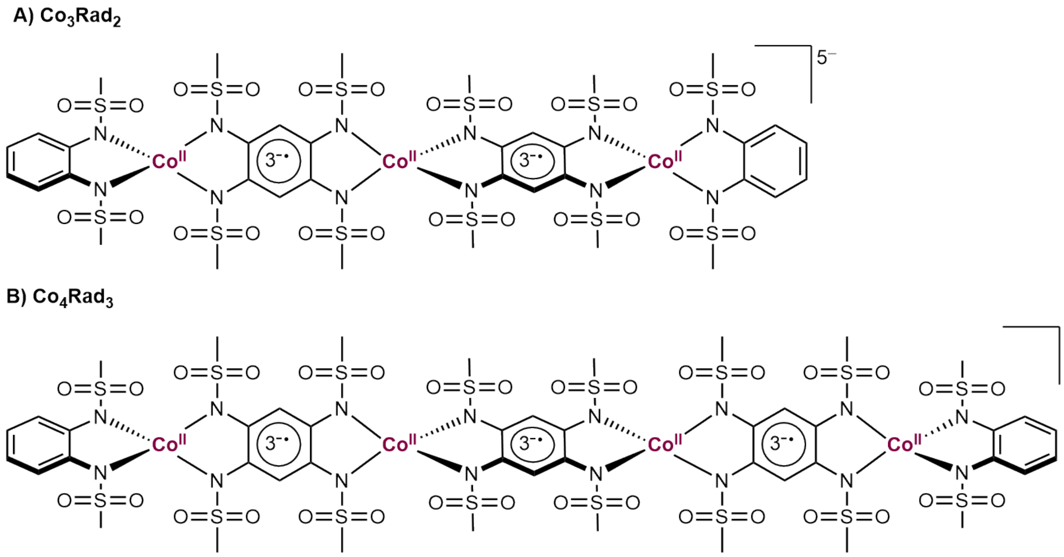

FIG. S10. **Cobalt chains molecular structure.** A) Molecular structure of $\mathbf{Co_3Rad_2}$. B) Mlecular structure of $\mathbf{Co_4Rad_3}$.

As evidenced by the similarity of relaxation profile between $\mathbf{Co_1}$ and $\mathbf{Co_2Rad}$, the local chemical environments generated by tmsab and bmsab ligands are very similar and lead to similar **D** values and spin-phonon coupling coefficients. To simulate the dynamics of the hypothetical $\mathbf{Co_3Rad_2}$ molecule (schematically reported in Fig. S10) we then approximate its spin Hamiltonian as

$$\hat{H} = \hat{H}_H + \hat{H}_{ZFS} + \hat{H}_Z \ , \tag{1}$$

where

$$\hat{H}_H = J(\,\vec{\mathbf{s}}_1 \cdot \vec{\mathbf{s}}_{b1} + \,\vec{\mathbf{s}}_2 \cdot \vec{\mathbf{s}}_{b1} + \,\vec{\mathbf{s}}_2 \cdot \vec{\mathbf{s}}_{b2} + \,\vec{\mathbf{s}}_3 \cdot \vec{\mathbf{s}}_{b2}) \ , \tag{2}$$

$$\hat{H}_{ZFS} = \vec{\mathbf{s}}_1 \cdot \mathbf{D} \cdot \vec{\mathbf{s}}_1 + \vec{\mathbf{s}}_2 \cdot \mathbf{D} \cdot \vec{\mathbf{s}}_2 + \vec{\mathbf{s}}_3 \cdot \mathbf{D} \cdot \vec{\mathbf{s}}_3 \tag{3}$$

and

$$\hat{H}_Z = \mu_B \vec{\mathbf{s}}_1 \cdot \mathbf{g} \cdot \vec{\mathbf{B}} + \mu_B \vec{\mathbf{s}}_2 \cdot \mathbf{g} \cdot \vec{\mathbf{B}} + \mu_B \vec{\mathbf{s}}_3 \cdot \mathbf{g} \cdot \vec{\mathbf{B}} + \mu_B \vec{\mathbf{s}}_{b1} \cdot \mathbf{g}_b \cdot \vec{\mathbf{B}} + \mu_B \vec{\mathbf{s}}_{b2} \cdot \mathbf{g}_b \cdot \vec{\mathbf{B}} \ . \tag{4}$$

Finally, each site is assumed to experience the same spin-phonon coupling. A logical extension follows for $\mathbf{Co_4Rad_3}$.

[1] Angelika B Boeer, Anne-Laure Barra, Liviu F Chibotaru, David Collison, Eric J L McInnes, Richard A Mole, Giovanna G Simeoni, Grigore A Timco, Liviu Ungur, Tobias Unruh, and Richard E P Winpenny, "A spectroscopic investigation of magnetic exchange between highly anisotropic spin centers," Angew. Chem. Int. Ed. **50**, 4007–4011 (2011).

[2] Frank Neese, "Importance of direct spin−spin coupling and spin-flip excitations for the zero-field splittings of transition metal complexes: a case study," J. Am. Chem. Soc. **128**, 10213–10222 (2006).

[3] Simon Suhr, David Hunger, Robert R. M. Walter, Andreas Köhn, Joris van Slageren, and Biprajit Sarkar, "Air-stable dinuclear complexes of four-coordinate Zn^{II} and Ni^{II} ions with a radical bridge: A detailed look at redox activity and antiferromagnetic coupling," Inorg. Chem. **63**, 6042–6050 (2024).
