# Supplementary material for: The Spin-Phonon Relaxation Mechanism of Single-Molecule Magnets in the Presence of Strong Exchange Coupling
Source: ACS Cent Sci. 2025 Mar 13;11(4):550–9. doi: 10.1021/acscentsci.4c02139 (PMC12022908; doi:10.1021/acscentsci.4c02139)
Supplement: Supplementary file 2 — oc4c02139_si_002.pdf [file oc4c02139_si_002.pdf]

oc-2024-02139g.R1

Name: Peer Review Information for "The spin-phonon relaxation mechanism of single-molecule magnets in the presence of strong exchange coupling"

First Round of Reviewer Comments

Reviewer: 1

Comments to the Author

In this manuscript Lunghi and coworkers report a full first-principle description of spin-phonon relaxation in an air-stable Co(II) dimer with both large single-ion anisotropy and strong exchange coupling through a delocalized radical. This detailed study, which extends to 3d exchange coupled systems the approach some of the authors used to mode,l by ab initio calculations, relaxation in single-ion complexes (see in particular ref. [20]), allow to draw some important conclusions and design rules for better performing Single Molecule Magnets (i.e., bistable magnetic molecules). On one side, they demonstrate that both Orbach and Raman relaxation mechanisms are mostly (but not exclusively) intra ground-state spin multiplet processes, and this is due to the strong exchange coupling in the specific system investigated. On the other hand, they clearly show that this would not hold when the exchange coupling becomes smaller than the single-ion zero-field splitting. Given the need to have large zero field splitting to observe slow relaxation of the magnetization, this translates into the first requirement of both a large zero field splitting and a large exchange coupling to observe bistability at high temeprature. While this is not a novel concept, being essentially the same strategy originally pursued by many research groups in the 90s and the first decade of the 00s, it is here derived ab initio for the first time, and it is to be considered as an important result. Even more relevant for the community is the insight the study provides into the control of Raman process, showing that, in perspective, increasing the nuclearity of the system could slow down Raman relaxation (the most detrimental one for application of these systems) by several orders of magnitude. My assessment of the manuscript is then extremely positive as for its scientific content. However, putting aside my personal scientific interests, I am doubtful as to its broad interest for researchers of other disciplines, as required for publication in ACS Central Science. A more specialized journal should probably be considered as more approriate for publication (Chemical Science, JACS Au).

At any rate the following points should be taken into account in a revised version (with particular attention to the first one):

- An important issue concerns the source of the relaxation data reported in Figure 2, to which the results of the calculations are compared. The experimental data are indicated as reported in ref.

26; however, inspection of ref. 26, which is only a preprint up to now (and since quite a long time) evidences that no dynamic data are reported there. The obvious question then arises as to the actual source of the reported data, that should be absolutely clarified. If these were not reported anywhere else previously, then a complete analysis (i.e., also showing the original dc relaxation and ac susceptibility curves, not only the extracted relaxation parameters) should be included here. I notice that in the ESI there are some issues with reference manager, since a question mark appears in place of – I guess - the same reference 26.

- Authors mention the fact that exchange coupling has been recognized as a key factor in controlling Quantum Tunneling of the magnetization for a long time, providing reference to the Nature 2002 work of Wernsdorfer and coworkers to substantiate their claim. However, the exchange coupling discussed in that paper for the control of QTM was of intermolecular type. It is thus not affecting the multiplet structure of the complexes but simply acts as an exchange bias field which shifts the QTM step from zero-field allowing a remanent magnetization to be observed. Here the effect of the exchange is quite different being crucial in determining the energy level structure, and I find that only referring to that work might be misleading. At least some discussion on this difference should be added.

- I find the sentence “We observe that the computed Orbach relaxation rates drastically drop as soon as phonons with energy larger than ∼ 180 are excluded from the simulation (see ESI)” is not completely clear and is only highlighting one side of the obtained result. What authors discuss here is a very important point, namely the fact that relaxation is taking place stepwise rather than via direct excitation to the second excited state. Thus, it is crucial to underline that the calculated relaxation time does not get longer if one removes phonons with energy in the range between 200 and 300 cm-1, which would not be the case if the relaxation was to take place in a single step to the second excited state. So this part might be expanded a little bit to stress this important take-home message, which is only reported in the Supplementary Material in the present version.

Reviewer: 2

Comments to the Author

Mondal et. al. present a computational methodology to obtain relaxation times in exchange coupled single molecule magnets (SMMs). This is a very timely work, offering a much-needed rationalisation of a complex problem that had remained unresolved till now, as well as new ways to keep improving the performance of SMMs.

The accuracy, and therefore worth of the proposed methodology, rely on the comparison to the experimental relaxation profile. However, the authors do not present the data used to obtain such

profile, which puts the whole study into question. I am convinced this has been an honest mistake and an unfortunate oversight, but I cannot recommend publication until this is fixed. See the specific comments on this and other aspects below.

On the magnetometry:

- In the SI, the authors mention that "The static magnetic properties of the dimer Co2Rad have been characterized in-depth before by magnetometry as well as by spectroscopic means[?].". However, no actual reference is given, making it impossible to assess whether this is true or not. I assumed this data could be found in reference 26 of the main text (Electronic structure of mononuclear and radical-bridged dinuclear cobalt (II) single-molecule magnets), but that was not the case. As already mentioned, since comparison to the experimental relaxation profile is key to validate the proposed methodology, one should be able to check the raw data and the fits employed to build the profile.

On the methods:

- More details on the electronic structure of the dimer should be presented. Why do the authors choose those particular number of states for different multiplicities? Have they done this by running a prior calculation with more states and checking the energy gaps?

- The active space of the dimer is a CAS(19,14), comprised of "the two 3d orbital sets and 4π orbitals of the radical bridge unit". Are the results, at least in terms of the energy splitting of the electronic states, dependent on the inclusion of the π* (antibonding) orbitals?

- The authors mention that "the inclusion of dynamic correlation is vital for accurate exchange couplings". Have they checked whether the NEVPT2 formalism would yield different results?

- The orbitals of the CASSCF calculations of the dimer should be shown in the SI.

- The authors mention that "Each molecular degree of freedom is sampled four times between ± 0.1 Å". What is the reason for this threshold and how can they be sure that this displacement is enough? Will this not over(under)sample low(high) energy phonons, leading to an uncompensated sampling?

- The authors employ an isotropic Heisenberg Dirac van-Vleck model spin Hamiltonian to extract the exchange coupling through the Lande formula. However, Co(II) ions are not isotropic. Could the authors comment on the effects that including anisotropic exchange interactions could have in the magnetic relaxation and why they are not considered in this work? Would the proposed computational approach allow one to include anisotropic exchange interactions?

- The authors discard the possibility of a magnetic interaction between the Co(II) ions (Eq. 8), but do not provide an explanation as to why. This should be clarified.

- Table 1 reports the effective exchange coupling constants assuming the Heisenberg Hamiltonian, which show significant variations depending on what spin states are considered. Could this not be caused by a non-negligible Co(II)-Co(II) interaction?

- The authors state that "The simulation of Kramers systems in zero external fields requires the use of the non-diagonal secular approximation [...] This is achieved by [...] orienting the molecular easy axis along the quantization z-axis and by applying a small magnetic field to break Kramers degeneracy". However, the data that the authors are reproducing have been obtained under a 0.2 T external magnetic field, which is far from small. Do the predicted relaxation times change if the calculations are done employing a 0.2 T field?

- The authors write that "the contribution of exchange coupling [...] has no contribution at the level of spin-phonon coupling". However, for those phonons affecting the most the distance between the centroid of the bridging aryl group and the Co(II), one would expect a non-negligible effect on the exchange, as the orbital overlap will change – this has been proven to be very relevant in organic radicals (Chem. Sci., 2015, 6, 2371-2381). To assess whether J really only enters into the static Hamiltonian, the authors might consider looping over the phonons, identifying the ones affecting this distance (or other parameter considered more relevant) and re-calculating the exchange interaction at those displaced geometries. The authors mention that by neglecting the derivatives of J in Eq. 2, results do not change, but I wonder whether this is an artefact arising from the relatively small applied distortion of 0.1 Å.

On the clusters:

- It is not clear how the authors propose to extend the system. They say that "the first ion is exchange-coupled to the second one, and the latter is exchange-coupled to the third." If that is the case, then Eq. 8 does no longer hold, as they are also including exchange interactions between the transition metal ions, and these systems cannot be compared to Co2rad.

- It also is not clear what the structure of these compounds would be. Assuming that the newly added ion is added to the left by changing two protons by two nitrogen atoms in the extreme aryl ring and then capped by a new aryl ring, because of the relative ~ 90 degrees angle between the bridging aromatic rings, the exchange interaction between the original two ions and the added one would be very different. Also, the main direction of the ZFS tensor of the third ion would likely be roughly orthogonal to those on the other ones in the molecular frame. However, the authors "assume the spin Hamiltonian parameters and spin-phonon coupling to be identical to the dimer". A clarification is needed here.

Other:

- No mention to the works of L. Ungur (https://doi.org/10.1002/ejic.202300598 & https://doi.org/10.1002/chem.202200227) and N. F. Chilton (J. Am. Chem. Soc. 2024, 146, 27, 18714–18721, J. Am. Chem. Soc. 2023, 145, 16, 8996–9002, 10.1126/science.abl5470) on the description of exchange coupling in anisotropic systems is made, which should be included.

- The authors write that “the present computations confirm a ferri-magnetic coupling of the two Co(II) centres via the radical bridge”. I find this sentence confusing for two reasons: i) ferrimagnetism arises from uncompensated antiferromagnetic interactions in a material, leading to an overall non-vanishing magnetic moment. However, as the authors discuss, the inversion symmetry of the compound enforces that both J interactions are the same. ii) Eq. 8 includes only an interaction between the Co(II) centre and the radical, no “coupling of the two Co(II) centres via the radical bridge”. This should be clarified/corrected.

- The colour code used in the bottom panel of Fig. 4 makes it hard to appreciate the changes that the authors are highlighting.

- On the “clusters of larger nuclearity”. The molecular structure of Co3Rad2 and Co4Rad3 should be presented.

- In Fig. 3, the caption reads “The y-axis reports the expectation value of the z component of the total spin angular momentum.” I believe this refers to the x-axis.

Author's Response to Peer Review Comments:

Response to the reviewers for:

The spin-phonon relaxation mechanism of single-molecule magnets in the presence of strong exchange coupling

Reviewer 1

Comment: My assessment of the manuscript is then extremely positive as for its scientific content. However, putting aside my personal scientific interests, I am doubtful as to its broad interest for researchers of other disciplines, as required for publication in ACS Central Science. A more specialized journal should probably be considered as more appropriate for publication (Chemical Science, JACS Au).

Reply: We thank the reviewer for the highly positive assessment of the importance of our scientific results. We must however kindly disagree with their conclusions in terms of the lack of broad appeal of our work, and indeed the journals suggested by the reviewer are also publishing works for a general readership.

Molecular magnetism is per se a highly multidisciplinary subject of interest for scientists across different fields. This goes from synthetic chemists to theoretical physicists, spectroscopists, and people working in magnetic resonance, magnetism, and quantum technologies. Our work provides

a roadmap for the design of robust single-molecule magnets which would not only surpass the current state-of-the-art single-ion magnets in terms of performance parameters, but would also exhibit stability under ambient conditions, making future efforts at device fabrication, for example through surface immobilization, a real possibility. This aspect would be highly challenging, if not impossible to achieve with current state-of-the-art lanthanide-based systems. As such the work has the potential to attain a large impact on both chemistry, materials and surface science, and device engineering.

The theoretical and numerical methods employed in this work are also highly interdisciplinary and provide quite a unique combination of spin relaxation theory, open quantum systems physics and quantum chemistry. Our key achievement of providing the first quantitative prediction of relaxation in exchange coupled compounds, not only provides a long-sought interpretation of relaxation in SMMs but it also paves the way to understanding relaxation in general spin systems. Overcoming single-spin relaxation theory will make it possible to investigate electro-nuclear systems of importance for NMR spectroscopy, exchange coupled radical probes used to sense macromolecules' dynamics, solid-state magnetic systems of relevance for condensed-matter physics and quantum science, to name a few.

As such, we believe that this contribution is very well placed to be published in a multi-disciplinary journal such as ACS Central Science.

We have now revised abstract, introduction and discussion sections to explicitly cover the broad relevance of our results beyond single-molecule magnets and make the aspects discussed above more evident to the general readership of the journal.

Comment: An important issue concerns the source of the relaxation data reported in Figure 2, to which the results of the calculations are compared. The experimental data are indicated as reported in ref. 26; however, inspection of ref. 26, which is only a preprint up to now (and since quite a long time) evidences that no dynamic data are reported there. The obvious question then arises as to the actual source of the reported data, that should be absolutely clarified. If these were not reported anywhere else previously, then a complete analysis (i.e., also showing the original dc relaxation and ac susceptibility curves, not only the extracted relaxation parameters) should be included here. I notice that in the ESI there are some issues with reference manager, since a question mark appears in place of – I guess - the same reference 26.

Reply: We apologise for this oversight. All relaxation data referenced in this work are included in the final version of ref. 26, now gone through the full editorial process at Nature Communications and is scheduled to be published soon. The data had been added to a second revision of the manuscript and neglected to update the preprint. The accepted version of the manuscript and its

supplementary information file are attached to this revision as additional info for review only. The reviewer will be able to observe the correspondence between the original experimental data and the ones reported in the present theoretical manuscript.

The magnetometry section from the ESI has been removed, as the present work does not report any novel experimental data. The broken references in the ESI have been fixed.

Comment: Authors mention the fact that exchange coupling has been recognized as a key factor in controlling Quantum Tunneling of the magnetization for a long time, providing reference to the Nature 2002 work of Wernsdorfer and coworkers to substantiate their claim. However, the exchange coupling discussed in that paper for the control of QTM was of intermolecular type. It is thus not affecting the multiplet structure of the complexes but simply acts as an exchange bias field which shifts the QTM step from zero-field allowing a remanent magnetization to be observed. Here the effect of the exchange is quite different being crucial in determining the energy level structure, and I find that only referring to that work might be misleading. At least some discussion on this difference should be added.

Reply: We agree with the reviewer that the situation is much more nuanced than what we suggested in the original manuscript. We have now added several references to the literature of both 3d and 4f SMMs as evidence that large intra-molecular exchange quenches QTM.

Comment: I find the sentence "We observe that the computed Orbach relaxation rates drastically drop as soon as phonons with energy larger than ∼ 180 are excluded from the simulation (see ESI)" is not completely clear and is only highlighting one side of the obtained result. What authors discuss here is a very important point, namely the fact that relaxation is taking place stepwise rather than via direct excitation to the second excited state. Thus, it is crucial to underline that the calculated relaxation time does not get longer if one removes phonons with energy in the range between 200 and 300 cm-1, which would not be the case if the relaxation was to take place in a single step to the second excited state. So this part might be expanded a little bit to stress this important take-home message, which is only reported in the Supplementary Material in the present version.

Reply: We have accordingly extended slightly the discussion of this point in the revised manuscript. We now highlight the importance of these different mechanisms in identifying the relevant phonons responsible for relaxation.

Reviewer 2

Comment: Mondal et. al. present a computational methodology to obtain relaxation times in exchange coupled single molecule magnets (SMMs). This is a very timely work, offering a much-needed rationalisation of a complex problem that had remained unresolved till now, as well as new ways to keep improving the performance of SMMs.

Reply: We thank the reviewer for appreciating the novelty and importance of our work and supporting its publication in ACS Central Science.

Comment: The accuracy, and therefore worth of the proposed methodology, rely on the comparison to the experimental relaxation profile. However, the authors do not present the data used to obtain such profile, which puts the whole study into question. I am convinced this has been an honest mistake and an unfortunate oversight, but I cannot recommend publication until this is fixed. See the specific comments on this and other aspects below.

Reply: We apologise for this oversight. As we have specified in the response to reviewer 1, all relaxation data referenced in this work are included in the final version of ref. 26, now gone through the full editorial process at Nature Communications and scheduled to be published soon. The relaxation data had been added to the second revision of that manuscript, which was however not updated at the preprint level by mistake. The accepted version of the manuscript and its supplementary information file are attached to this revision as additional info for review only. The reviewer will be able to observe the correspondence between the original experimental data and the ones reported in the present theoretical manuscript.

Comment: In the SI, the authors mention that "The static magnetic properties of the dimer Co2Rad have been characterized in-depth before by magnetometry as well as by spectroscopic means[?].". However, no actual reference is given, making it impossible to assess whether this is true or not. I assumed this data could be found in reference 26 of the main text (Electronic structure of mononuclear and radical-bridged dinuclear cobalt (II) single-molecule magnets), but that was not the case. As already mentioned, since comparison to the experimental relaxation profile is key to validate the proposed methodology, one should be able to check the raw data and the fits employed to build the profile.

Reply: The reference in the ESI has been fixed, now pointing to ref. 26. We kindly refer the reviewer to the previous reply in regard to the availability of the experimental data. The section on magnetometry has been removed from the ESI as this work does not report any novel experimental data beyond those included in the final version of ref. 26.

Comment: Why do the authors choose those particular number of states for different multiplicities? Have they done this by running a prior calculation with more states and checking the energy gaps?

Reply: Each Co(II) ion features 2 low-lying quartet states (discussed in more detail in Ref. 26 and previous literature). Each of these states can couple via the unpaired electron of the bridge giving a spin ladder with 2 doublets, 2 quartets, 2 sextets and 1 octet. As there are two low-lying quartets per Co(II) ion, there are 4 such ladders giving the mentioned states. We have (in the course of Ref. 26) also done computations with more states, which confirm a gap. We give a short hint on that in the main text and further explanations in the supporting information.

Comment: The active space of the dimer is a CAS(19,14), comprised of "the two 3d orbital sets and 4π orbitals of the radical bridge unit". Are the results, at least in terms of the energy splitting of the electronic states, dependent on the inclusion of the π* (antibonding) orbitals?

Reply: The design of active spaces for systems of the present size is challenging and "converged" active spaces are difficult to achieve and to prove. We rely here on the experience made in Ref. 26 and for similar systems (e.g. the analogous Ni compound). We have now also checked a smaller active space that only includes the singly occupied orbital on the ligand. This leads to a slightly smaller prediction for J (374 cm-1), but confirms that our results are not heavily active space dependent.

Comment: The authors mention that "the inclusion of dynamic correlation is vital for accurate exchange couplings". Have they checked whether the NEVPT2 formalism would yield different results?

Reply: We included dynamic correlation using the CASPT2 formalism and the statement above is substantiated by the comparison to CASCI energy levels (detailed results are also provided as a data collection in the provided spreadsheet files). As the molecule is rather large, for the CASPT2 computation a pair-natural-orbital space-based approach is required (PNO-CASPT2), which narrows down the applicable software packages (here: Molpro). NEVPT2 is an alternative option, but a large-scale implementation is only available in a different program package (ORCA), which we have not tried yet. A full benchmark of computational methods is beyond the scope of the current work, but it certainly represents a very interesting and important avenue for future investigation.

Comment: The orbitals of the CASSCF calculations of the dimer should be shown in the SI.

Reply: We provide the requested information in the ESI.

Comment: The authors mention that “Each molecular degree of freedom is sampled four times between ± 0.1 Å”. What is the reason for this threshold and how can they be sure that this displacement is enough? Will this not over(under)sample low(high) energy phonons, leading to an uncompensated sampling?

Reply: The numerical differentiation strategy used here has been benchmarked over almost a decade of work in computing spin-phonon coupling by some of the authors and 4 steps with displacement +/- 0.05 and +/- 0.1 Angstrom for all Cartesian degrees of freedom is a rather standard set of values. We are now reporting a couple of examples for both hard/soft degrees of freedom, e.g. one atom from the first coordination shell and one hydrogen atom to reassure the reviewer that this strategy makes it possible to perfectly capture the profile of D and J along atomic displacements.

Comment: The authors employ an isotropic Heisenberg Dirac van-Vleck model spin Hamiltonian to extract the exchange coupling through the Lande formula. However, Co(II) ions are not isotropic. Could the authors comment on the effects that including anisotropic exchange interactions could have in the magnetic relaxation and why they are not considered in this work? Would the proposed computational approach allow one to include anisotropic exchange interactions?

Reply: Anisotropic exchange occurs only due to (interatomic) spin-orbit (or spin-spin) coupling and hardly exceeds a wavenumber for 3d elements (the two Co ions are several Å away from each other). In view of the over 400 cm^{-1} isotropic exchange, anisotropic exchange is expected to have a very small effect. Moreover, the proposed spin Hamiltonian fits computed (spin-orbit coupled) energy levels very well and also fits experimental values (see also Ref. 26) adding further parameters will likely result in overfitting. In reference 10.1021/ acs.inorgchem.4c00351 some of the authors have investigated anisotropic exchange for a homologous Ni complex and did not find any evidence for anisotropic exchange, both from fitting experimental data (SQUID and ESR) or the electronic levels (after introducing spin-orbit coupling). We comment on this issue in the main text (Results, paragraph

“Electronic structure and magnetic interactions”) and in the ESI (section “Spin Hamiltonian values”)

Comment: The authors discard the possibility of a magnetic interaction between the Co(II) ions (Eq. 8), but do not provide an explanation as to why. This should be clarified.

Reply: We added a short note concerning this term in the main text (see also reply to next comment).

Comment: Table 1 reports the effective exchange coupling constants assuming the Heisenberg Hamiltonian, which show significant variations depending on what spin states are considered. Could this not be caused by a non-negligible Co(II)-Co(II) interaction?

Reply: We have tried this, but clearly assuming such an interaction does not lead to an improved fit. We added a table in the ESI (S4) to demonstrate this.

Comment: The authors state that "The simulation of Kramers systems in zero external fields requires the use of the non-diagonal secular approximation [...] This is achieved by [...] orienting the molecular easy axis along the quantization z-axis and by applying a small magnetic field to break Kramers degeneracy". However, the data that the authors are reproducing have been obtained under a 0.2 T external magnetic field, which is far from small. Do the predicted relaxation times change if the calculations are done employing a 0.2 T field?

Reply: As long as the applied field is along the easy axis of the SMM, it has no effect on the relaxation rate. The field in experiments, if it is not excessively large (less than 0.6 T as shown in the ESI of the attached version of ref. 26), has no effect on spin phonon relaxation but quenches QTM and it is key to making simulations and experiments compatible. This is discussed in detail in Lunghi Sci.

Adv. 8 (31), eabn7880 2022. The revised manuscript now includes a comment on the effect of an external field on both exp and simulated relaxation times.

Comment: The authors write that "the contribution of exchange coupling [...] has no contribution at the level of spin-phonon coupling". However, for those phonons affecting the most the distance between the centroid of the bridging aryl group and the Co(II), one would expect a non-negligible effect on the exchange, as the orbital overlap will change – this has been proven to be very relevant in organic radicals (Chem. Sci., 2015, 6, 2371-2381). To assess whether J really only enters into the static Hamiltonian, the authors might consider looping over the phonons, identifying the ones affecting this distance (or other parameter considered more relevant) and re-calculating the exchange interaction at those displaced geometries. The authors mention that by neglecting the derivatives of J in Eq. 2, results do not change, but I wonder whether this is an artefact arising from the relatively small applied distortion of 0.1 Å.

Reply: The derivatives of J are indeed computed as different from zero as the reviewer suggests, but their contribution to spin relaxation is negligible with respect to the effect of the derivatives of the zero-field splitting. This can be traced back to the nature of the spin operator multiplying dJ/dq. The scalar product between spin operators does not mix spin states with different M_S, hence contributing little to spin relaxation. We have now updated the paragraph to make this clearer.

Comment: It is not clear how the authors propose to extend the system. They say that "the first ion is exchange-coupled to the second one, and the latter is exchange-coupled to the third." If that is the case, then Eq. 8 does no longer hold, as they are also including exchange interactions between the transition metal ions, and these systems cannot be compared to Co2rad.

Reply: Our wording was not sufficiently precise. We propose a chain of Co-Rad-Co-Rad-Co, where three Co(II) are coupled by two radical bridges. This is a straightforward extension of Eq. 8. and an approximate estimation of its relaxation does not require any additional ab initio calculation. We have now rephrased the text to make this clear and added a simple scheme in the inset of Fig. 6. The nature of the spin Hamiltonian used for this simulation is now detailed in the ESI.

Comment: It also is not clear what the structure of these compounds would be. Assuming that the newly added ion is added to the left by changing two protons by two nitrogen atoms in the extreme aryl ring and then capped by a new aryl ring, because of the relative ~ 90 degrees angle between the bridging aromatic rings, the exchange interaction between the original two ions and the added one would be very different. Also, the main direction of the ZFS tensor of the third ion would likely be roughly orthogonal to those on the other ones in the molecular frame. However, the authors "assume the spin Hamiltonian parameters and spin-phonon coupling to be identical to the dimer". A clarification is needed here.

Reply: We kindly refer the reviewer to the previous answer. We also would like to clarify that as mentioned in the results section of the manuscript, the easy axis lies along the Co-Co direction. Extending the molecule in a chain would thus maintain the easy axis of the Co ions parallel to each other, making the use of the same spin Hamiltonian for each Co a good approximation.

Comment: No mention to the works of L. Ungur (https://doi.org/10.1002/ejic.202300598 & https://doi.org/10.1002/chem.202200227) and N. F. Chilton (J. Am. Chem. Soc. 2024, 146, 27, 18714–18721, J. Am. Chem. Soc. 2023, 145, 16, 8996–9002, 10.1126/science.abl5470) on the description of exchange coupling in anisotropic systems is made, which should be included.

Reply: We have added several additional references to the manuscript to illustrate the state of the art in calculating exchange coupling in SMMs. The list includes a balanced sampling of 20 years of literature on this topic as well as some of the references proposed by the reviewer.

Comment: The authors write that "the present computations confirm a ferri-magnetic coupling of the two Co(II) centres via the radical bridge". I find this sentence confusing for two reasons: i) ferrimagnetism arises from uncompensated antiferromagnetic interactions in a material, leading to an overall non-vanishing magnetic moment. However, as the authors discuss, the inversion symmetry of the compound enforces that both J interactions are the same. ii) Eq. 8 includes only an interaction between the Co(II) centre and the radical, no "coupling of the two Co(II) centres via the radical bridge". This should be clarified/corrected.

Reply: Ferrimagnetic referred to the fact that the two Co(II) centers couple antiferromagnetically to the radical. Hence the ground state is characterized by a +3/2 – -1/2 – +3/2 coupling, effectively leading to a total 5/2 (but not 7/2) spin. We have updated this paragraph to avoid using the term ferrimagnetic but more precisely refer to the parallel alignment of the spin of the Co ions due to the antiferromagnetic coupling with the radical bridge.

Comment: The colour code used in the bottom panel of Fig. 4 makes it hard to appreciate the changes that the authors are highlighting.

Reply: We have done our best to improve the clarity of the graphics.

Comment: On the "clusters of larger nuclearity". The molecular structure of Co3Rad2 and Co4Rad3 should be presented.

Reply: A ChemDraw schematic has now been added to the supplementary information, together with the spin Hamiltonian used to generate the simulations.

Comment: In Fig. 3, the caption reads "The y-axis reports the expectation value of the z component of the total spin angular momentum." I believe this refers to the x-axis.

Reply: We thank the reviewer for bringing this typo to our attention.

oc-2024-02139g.R2

Name: Peer Review Information for "The spin-phonon relaxation mechanism of single-molecule magnets in the presence of strong exchange coupling"

Second Round of Reviewer Comments

Reviewer: 1

Comments to the Author

In this revised version, the authors convincingly answered the specific points I raised in my previous report. The expansion of the Abstract and of the Introduction also helps the general reader of the Journal to understand the relevance of the results presented here beyond the molecular magnetism community.

I am then glad to suggest acceptance of the manuscript in its present version. I only suggest here a very minor correction which might well be included at the proof stage:

-Just repeat the call to ref. 39 in the caption to figure 2 to let the reader know immediately where the original experimental data can be found.

Author's Response to Peer Review Comments:

We thank the reviewer for supporting the publication of our manuscript and recognizing the broad importance of our contribution.
